# Comparison of the inhibitory effect of tocilizumab and etanercept on the progression of joint erosion in rheumatoid arthritis treatment

**DOI:** 10.1038/s41598-022-22152-w

**Published:** 2022-10-20

**Authors:** Shinya Hayashi, Tsukasa Matsubara, Toshihisa Maeda, Koji Fukuda, Keiko Funahashi, Marowa Hashimoto, Ken Tsumiyama, Tomoyuki Kamenaga, Yoshinori Takashima, Tomoyuki Matsumoto, Shotaro Tachibana, Ryosuke Kuroda

**Affiliations:** 1 Department of Orthopaedic Surgery, Kobe University Graduate School of Medicine, 7-5-1 Kusunoki-cho, Chuo-ku, Kobe, 650-0017 Japan; 2 Department of Orthopaedic Surgery, Matsubara Mayflower Hospital, Kato, Japan; 3 Research Institute of Joint Diseases, Kobe, Japan

**Keywords:** Rheumatoid arthritis, Musculoskeletal system

## Abstract

We compared the efficacy of tocilizumab and etanercept in inhibiting radiographic progression of joint destruction in rheumatoid arthritis. Overall, 187 patients treated with etanercept or tocilizumab were selected. To adjust for baseline patient characteristics between the tocilizumab and etanercept treatment groups, a propensity score matching was performed. Radiographic progression of joint destruction was compared between patients treated with tocilizumab or etanercept. Clinical disease activity index (CDAI) and modified health assessment questionnaire (mHAQ) scores at the administration of biologic treatment and after 12 months of tocilizumab and etanercept therapy were measured and compared to radiographical parameters between the groups. Levels of C-reactive protein (CRP), matrix metalloproteinase-3 (MMP-3), CDAI, and mHAQ scores improved after 12 months of treatment in the two groups. Proportion of patients with no Sharp erosion score progression was significantly higher with tocilizumab treatment than with etanercept treatment (*p* = 0.032). Multivariate analysis demonstrated that Sharp erosion score was significantly associated with baseline CDAI (odds ratio, 1.05; 95% confidence interval, 1.003–1.099, *p *= 0.037). Tocilizumab treatment suppressed joint erosion progression compared to etanercept, and the progression correlated with baseline CDAI.

## Introduction

Rheumatoid arthritis (RA) is an autoimmune disease characterised by chronic inflammation of the synovial lining of the joint^1^ and is characterized by progressive joint destruction and systemic complications^2^. Radiographic damage is one of the most critical outcomes of RA, and effective therapy reduces the progression of joint destruction^3^. The destruction of joints is due to overproduction of pro-inflammatory cytokines, including tumour necrosis factor-alpha (TNF-α), interleukins (IL) -6 and -17, and macrophage colony-stimulating factor (GM-CSF) from immune cells in the synovium^4^. The drugs that inhibit cytokine productions have been investigated as possible treatments to halt the progression of joint destruction in RA^5^.

IL-6 plays a significant role in the pathogenesis of RA and disease activity^6^. The expression of serum IL-6 and IL-6 receptor is correlated with inflammation, clinical signs and symptoms, and radiographic signs of joint destruction^7,8^. IL-6 affects the function of neutrophils, T cells, B cells, monocytes, and osteoclasts, which are highly activated in RA. IL-6 also affects the hepatic acute phase response, which is a key feature of RA^9^. As a key regulator in osteoclast differentiation, IL-6 may promote erosive joint changes by activating osteoclast formation and accelerating bone resorption^8^. Several clinical studies have demonstrated that tocilizumab treatment (IL-6 inhibitor) is effective in inhibiting bone erosion^10–14^. The SAMURAI study reported that tocilizumab treatment is more effective in preventing the progression of bone erosion than conventional treatment with disease-modifying antirheumatic drugs (DMARDs); however, no significant difference in joint space narrowing (JSN) was observed between the two groups at 28 weeks^10^. The FUNCTION study reported that methotrexate (MTX)-only treatment caused greater progression of bone erosion than treatment with tocilizumab and MTX, but no significant difference in JSN was observed at 52 weeks^12,13^. Hashimoto and colleagues also reported that tocilizumab monotherapy was more effective in reducing radiographical progression of bone erosion in patients presenting with risk factors for rapid joint destruction^14^.

TNF inhibitors also play a role in the inhibition of radiographic progression of joint destruction^15,16^. TNF-α induces the secretion of multiple proinflammatory cytokines such as IL-1, -6, and -8; GCS-F; and dickkopf (DKK)-1, which regulate the Wnt pathway^17,18^. Previous reports showed that TNF inhibitors decrease serum DKK-1 levels in patients with RA^18^, and DKK-1 levels correlated with extent of the radiographic joint destruction^19^. Therefore, it is important to understand the role of Wnt pathway molecules including DKK-1 and sclerostin; however, data on the in vivo effect of TNF-α inhibitor on bone loss in patients with RA are limited^20,21^. The TEMPO study reported that mean changes in radiographic progression were significantly lower for patients receiving a combination of etanercept and MTX or etanercept monotherapy than for those receiving MTX monotherapy^15^. Mean changes from baseline in erosion scores of modified total Sharp score (mTSS) for the combination and etanercept groups were significantly lower than those in the MTX group; however, the significance of this finding has not been analysed^15^. These reports indicate that the IL-6 inhibitor or the TNF inhibitor reduced radiographic progression of joint destruction. However, none of the studies have compared the inhibitory effect of the IL-6 inhibitor and TNF inhibitor on the radiographic progression of joint destruction. Tocilizumab and etanercept are commonly used in the treatment of RA as biological drugs for inhibition of IL-6 and TNF, respectively^22,23^.

Therefore, the present study aimed to compare the efficacy of tocilizumab and etanercept in inhibiting the radiographic progression of joint destruction.

## Materials and Methods

### Ethics statement and patient consent

This study complies with the Declaration of Helsinki, and study protocols were approved by the ethics committee of the Research Institute of Joint Disease Kobe and Kobe University Graduate School of Medicine. All participants provided informed consent for participation.

### Patient selection

This was a retrospective cohort study. Medical records of 202 patients with RA who were treated with a TNF inhibitor (etanercept) or an IL-6 inhibitor (tocilizumab) at Matsubara Mayflower Hospital and Kobe University Hospital between October 2004 and September 2020 were analysed. All patients included in the study were qualified according to the 1987 American College of Rheumatology RA criteria^24^. Only patients who were treated with etanercept (96 patients) or tocilizumab (121 patients) for at least 12 months were included in this study. However, patients who had a treatment overlap between etanercept and tocilizumab were excluded. Finally, 187 patients were included and were divided into two groups with etanercept treatment (81 patients) or tocilizumab treatment (106 patients).

### Clinical evaluation

Data were collected on variables such as age, duration of RA, MTX dose (mg/week), glucocorticoid dose (mg/day), C-reactive protein (CRP) level, and matrix metalloproteinase-3 (MMP-3) at the time of introduction of the tocilizumab or etanercept therapy. The duration of RA was determined between the time of RA diagnosis and administration of tocilizumab or etanercept. Data of mHAQ^25^ and CDAI^26^ were obtained as clinical outcomes at the initiation of treatment and after 12 months of tocilizumab or etanercept therapy.

### Radiographic evaluation

The x-rays were taken at the start of the drug treatment and also after 12 months. Radiographs of the hands and feet were assessed according to the Sharp method^27^. The scores for 187 radiographs (from 187 patients) were determined by two experienced rheumatologists who were blinded to the clinical data. Sixteen and six areas were considered for assessing erosions and JSN for the hands and feet, respectively. The maximum erosion score of the hands and wrists was 160 and that of the feet was 120 (maximum total erosion score: 280). Accordingly, the maximum JSN score of the hands and wrists was 120 and that of the feet was 48 (maximum total JSN score: 168). The sum of the erosion and JSN scores was calculated as the total Sharp/van der Heijde score (mTSS) (maximum: 448)^28^. The average score of the readings was used as the radiographic score. The proportion of patients showing no radiographic progression was determined using thresholds set at changing from the baseline mTSS (delta mTSS), changing from the baseline Sharp erosion score (delta Sharp erosion score), or changing from the baseline Sharp JSN score (delta JSN) ≤ 0, ≤ 0.5, and ≤ the smallest detectable change (SDC). The SDC values at each timepoint were estimated with the SD of the differences between delta mTSS, delta erosion, or delta JSN assigned by the two blinded image assessors^29^.

### Propensity score‑matched analysis

To adjust for baseline patient characteristics between the tocilizumab and etanercept treatment groups, a propensity score matching was performed^30^. Propensity scores were calculated from logistic regression models. In the present study, patient characteristics data (sex, age, RA duration, CDAI, mHAQ, first bDMARDs or more, CRP levels, MTX dose, and glucocorticoid dose) and radiographic scores (Total Sharp score) at biological drug administration were used to calculate a propensity score; matching of one patient with tocilizumab treatment to another patient with etanercept treatment with the same propensity score was performed. The pairing was achieved with the caliper tolerance of 20% of standard deviation of propensity score, and a random selection was made among the patients with the same propensity score. Finally, 38 pairs of patients were matched in two groups. The patient characteristics before and after propensity matching are shown in Table 1.

**Table 1:** Patient characteristics before and after propensity score matching.

Patient number 187	Before matching			After matching		
	Etanercept (81)	Tocilizumab (106)	*p*-value	Etanercept (38)	Tocilizumab (38)	*p*-value
Sex female/ male	65/ 16	81/ 25	0.328	12/26	14/ 24	0.809
Age	51.3 ± 13.7	62.8 ± 12.5	< 0.001	57.0 ± 13.1	56.0 ± 12.4	0.741
RA duration	3.6 ± 3.7	8.6 ± 9.2	< 0.001	4.2 ± 4.3	4.7 ± 6.7	0.684
First bDMARDs	64	53		26	23	
Second or more bDMARDs	17	53	< 0.001	12	15	0.632
CDAI	20.3 ± 12.6	22.9 ± 13.1	0.210	22.3 ± 12.9	18.3 ± 9.7	0.127
mHAQ	0.6 ± 0.7	0.6 ± 0.6	0.731	0.7 ± 0.8	0.6 ± 0.5	0.172
CRP	1.9 ± 2.2	2.8 ± 2.6	0.024	2.5 ± 2.5	2.2 ± 2.1	0.606
MMP3	240 ± 257	277 ± 202	0.300	329 ± 327	289 ± 224	0.529
MTX dose (mg/week)	3.9 ± 5.1	3.9 ± 4.2	0.958	3.7 ± 4.0	3.3 ± 4.3	0.310
Glucocorticoid dose (mg/day)	2.0 ± 2.6	2.6 ± 2.8	0.133	2.9 ± 2.9	2.4 ± 2.4	0.356
Total Sharp score	20.5 ± 32.5	17.8 ± 52.1	0.701	26.1 ± 40.1	27.6 ± 74.0	0.913

*bDMARDs* biological disease-modifying antirheumatic drugs, *CRP* C-reactive protein, *MMP-3* matrix metalloproteinase-3, *CDAI* clinical disease activity index (CDAI), *mHAQ* modified health assessment questionnaire, *MTX* methotrexate.

### Statistical analysis

Demographic and clinical characteristics of patients treated with tocilizumab or etanercept are provided in Tables 1, 2, 3, 4, and 5. All data are expressed as mean ± standard deviation unless otherwise indicated. Patients' background characteristics and radiographic progression between the two groups were compared using Mann–Whitney U test and paired t-test before and after propensity matching, respectively (Table 1). To assess the improvement in laboratory parameters (CRP levels and MMP-3) and clinical scores (mHAQ and CDAI), we compared values at biological drug administration and those after 12 months using paired t-test (Table 2). In addition, to assess the radiographic progression and risk factor of Sharp erosion score progression for treatment with tocilizumab and etanercept, Fisher’s exact test for nominal variables were performed (Tables 3 and 5). Correlations between clinical parameters and Sharp erosion score progression were analyzed using Pearson’s correlation value (Tables 4). Additionally, we performed a multivariate analysis to test the association between CDAI, mHAQ, or treatment with tocilizumab or etanercept and radiographic progression of Sharp erosion score (Table 6). Odds ratios (ORs) and 95% confidence intervals (CIs) were calculated for multivariate analysis. Data were analysed using SPSS, version 19 J (IBM Japan, Tokyo, Japan).

**Table 2:** Comparison of laboratory data and clinical outcome between baseline and 12 months after drug administration.

	Baseline	12 months	*p*-value
**ETN**			
CRP	2.5 (2.5)	0.4 (0.6)	< 0.001
MMP-3	255 (282)	169 (139)	< 0.001
CDAI	22 (13)	7 (7)	< 0.001
mHAQ	0.7 (0.8)	0.2 (0.5)	< 0.001
**TCZ**			
CRP	2.2 (2.1)	0.1 (0.3)	< 0.001
MMP-3	316 (307)	77 (49)	0.001
CDAI	18 (10)	8 (6)	< 0.001
mHAQ	0.6 (0.5)	0.2 (0.3)	< 0.001

Data represent as mean (standard deviation).

*ETN* etanercept, *TCZ* tocilizumab, *CRP* C-reactive protein, *MMP-3* matrix metalloproteinase-3, *CDAI* clinical disease activity index (CDAI); *mHAQ* modified health assessment questionnaire.

**Table 3:** Proportion of patients with no radiographic progression defined by delta mTSS ≤ 0, ≤ 0.5, and ≤ SDC.

		Etanercept	Tocilizumab	*p*-value
Delta mTSS(%)	≦ 0	47.4	50.0	1.000
	≦ 0.5	57.9	60.5	1.000
	≦ SDC	71.1	60.5	0.919
Delta mTSS (joint narrow) (%)	≦ 0	63.2	50.0	0.161
	≦ 0.5	73.7	65.8	0.449
	≦ SDC	73.7	76.3	1.000
Delta mTSS (erosion) (%)	≦ 0	63.2	86.8	0.032
	≦ 0.5	63.2	89.5	0.014
	≦ SDC	63.2	86.8	0.032

*mTSS* modified total Sharp score, *SDC* smallest detectable change.

**Table 4:** Correlation of clinical parameters at drug administration and yearly progression of erosion score at 12 months after drug administration.

	Correlation coefficient	*p*-value
Age	0.10	0.371
RA duration	− 0.07	0.574
CRP	0.11	0.325
MMP-3	0.01	0.895
CDAI	0.37	< 0.001
mHAQ	0.30	0.008
Glucocorticoid dose (mg/day)	0.06	0.613

*RA* rheumatoid arthritis, *CRP* C-reactive protein, *MMP-3* matrix metalloproteinase-3, *CDAI* clinical disease activity index (CDAI), *mHAQ* modified health assessment questionnaire.

**Table 5:** Comparison at drug administration between yearly progression of Sharp erosion score≦ 0, 0.5 group and > 0, 0.5 group.

Patients number (n = 76)	Sharp erosion < 0 (n = 57)	Sharp erosion > 0 (n = 19)	*p*-value
First biological drug / second or more	36/21	13/6	0.786
Etanercept/ tocilizumab	24/33	14/5	0.032
Male/female	13/43	5/14	0.765

	Sharp erosion < 0.5 (n = 59)	Sharp erosion > 0.5 (n = 17)	
First biological drug/second or more	37/22	12/5	0.774
Etanercept/tocilizumab	24/35	14/3	0.005
Male/ female	13/46	5/12	0.531

**Table 6:** Results of the multivariate analysis for predictive factors of progression of bone erosion score.

Predictive factor	Odds ratio (95% CI)	*p*-value
CDAI at baseline	1.05 (1.003–1.099)	0.037

*CI* confidence interval, *CDAI* Clinical disease activity index.

## Results

### Treatment with biological drugs improved laboratory parameters and clinical outcomes

Laboratory data and clinical outcomes of our study are provided in Table 2. Treatment with etanercept or tocilizumab for 12 months significantly improved laboratory data (CRP levels and MMP-3) and clinical outcomes (mHAQ and CDAI) in both treatment groups (Table 2).

### Radiographic progression was different between etanercept and tocilizumab treatment group

**Figure 1:**
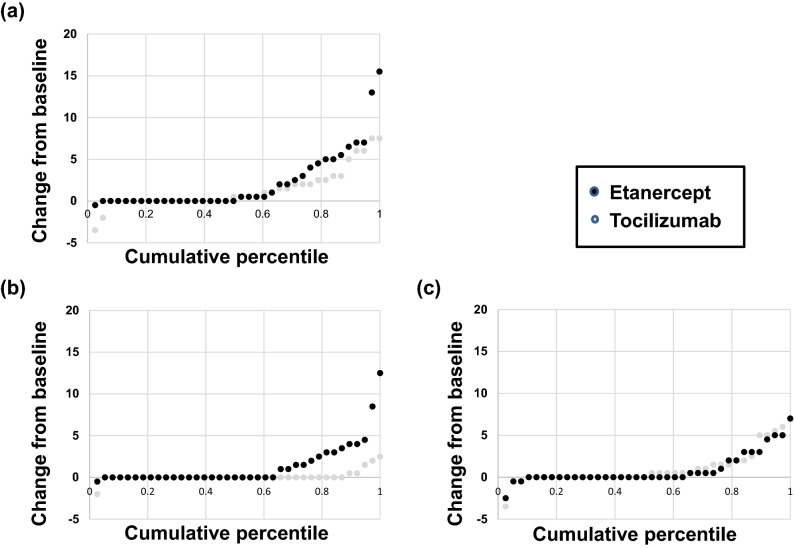
Radiographic progression in structural joint damage was evaluated using cumulative distribution of mTSS change from baseline at one year. Black dots represent the progression of etanercept and gray dots represents the progression of tocilizumab (**a**) delta mTSS (**b**) delta erosion (**c**) delta JSN.

Radiographic progression in structural joint damage was evaluated using cumulative distribution of mTSS, Sharp erosion score, and Sharp joint space narrow score change from baseline to 12 months after drug administration (Fig. 1). A proportion of patients with no radiographic progression defined by delta mTSS (delta mTSS), delta Sharp JSN (delta JSN), and delta Sharp erosion score (delta erosion) are shown in Table 3. Significant differences in progression for Sharp erosion score using thresholds of delta erosion ≤ 0, ≤ 0.5, and ≤ SDC were found between the tocilizumab and etanercept groups (Table 3). However, no significant difference was found in progression based on delta mTSS and delta JSN (Table 3).

### Predictive factors for radiographic progression of erosion for treatment with biological drugs

We have analyzed the predictive factors including age, RA duration, baseline CRP, MMP-3, CDAI, mHAQ, glucocorticoid dose, first biological drug or more, etanercept or tocilizumab treatment, and sex of patients in relation with the progression of Sharp erosion score (Tables 4 and 5). The baseline CDAI and mHAQ were significantly correlated with delta erosion (Table 4), and the proportion of patients with delta erosion differed significantly in the etanercept or tocilizumab treatment groups (Table 5). Predictive factors may be dependent on multiple confounders. Therefore, the significant predictive factors detected by univariate analysis (baseline CDAI, mHAQ, and treatment with etanercept or tocilizumab) were used as covariates for multivariate analysis. We demonstrated that the radiographic delta erosion was significantly associated with baseline CDAI (OR, 1.05; 95% CI, 1.003–1.099, *p *= 0.037) (Table 6).

## Discussion

In the present study, we demonstrated that the radiographic progression of joint erosion with tocilizumab treatment was significantly lower than that with etanercept treatment for RA. Multivariate analysis demonstrated that radiographic progression of joint erosion was associated with the baseline CDAI.

Bone erosion depends on osteoclast formation in the joint^31^. Monocyte/macrophage lineages infiltrate into inflamed joints and differentiate into osteoclasts, which play an important role in bone resorption^31^. Receptor activation of the NF-κB ligand (RANKL) is the primary factor in osteoclast differentiation and promotes osteoclast differentiation mainly through controlling gene expression by activating its receptor, RANK^32^. It is conceivable that drugs that suppress joint inflammation also inhibit monocyte/macrophage infiltration and, consequently, osteoclast formation.

IL-6 is considered a key molecule in driving osteoclastogenesis and bone resorption in RA^8,33^. RANKL is induced by IL-6 in mesenchymal cells, and IL-6 also influences T lymphocytes to support osteoclastogenesis^34,35^. Interaction of IL-6 with IL-6R on osteoclast precursors directly influences osteoclast formation without RANKL signalling^36^. Consequently, IL-6R directly affects osteoclast formation independent of its anti-inflammatory effects^37^. TNF-α also stimulates osteoclastogenesis through osteoclast precursors primed by a sufficient level of RANKL^38^. TNF-α binds to its receptors on stromal or osteoblastic cells and enhances RANKL expression^39^. Thus, TNF-α plays a pivotal role in enhancing the pathogenesis of inflammatory bone erosion in the presence of RANKL. These differences in mechanism underlying the stimulation of osteoclastogenesis may be one of the reasons that explain our finding that the inhibitory effect on progression of joint erosion was higher with the IL-6 inhibitor than with the TNF-α inhibitor.

Several clinical trials demonstrated that seropositivity for anti-citrullinated protein (CCP) antibodies, rheumatoid factor, inflammatory markers including CRP, baseline erosion, swollen joint count, and persistent disease activity were associated with rapid joint destruction in patients with RA^40,41^. A recent report showed that patients who achieve clinical remission within six months, as defined based on the DAS28-CRP, SDAI, CDAI, and ACR/EULAR Boolean criteria, have a high likelihood of remaining free of radiographic structural progression^42^. Welsing et al. investigated the longitudinal relationship between inflammatory disease activity and radiologic progression and demonstrated that high disease activity at base line causes progression of radiologic damage^43^. The results of these studies support our findings of multivariate analysis that the radiographic progression of joint erosion was associated with the baseline CDAI.

The limitations of this study are as follows: First, the sample sizes in subgroups of the cohort were not large, and power was limited. Therefore, the data must be accumulated and re-analysed in the future. Second, the database used in the analysis was obtained retrospectively. Third, the present study focused on results for 12 months after drug administration. Long-term outcomes with more than 12 months are yet to be elucidated. Fourth, the study only enrolled patients from two hospitals in one country therefore our results may be subject to geographical bias.

## Conclusions

We demonstrated that tocilizumab is more effective in suppressing radiographic progression of joint erosion compared to etanercept, and the radiographic progression of joint erosion was associated with baseline CDAI.

## Data availability

All data generated or analyzed during this study are included in this published article.
